# Use of Complementary and Alternative Medicines (CAMs) among type 2 diabetes patients in Sri Lanka: a cross sectional survey

**DOI:** 10.1186/1472-6882-14-374

**Published:** 2014-10-04

**Authors:** Arjuna B Medagama, Ruwanthi Bandara, Rajitha A Abeysekera, Buddhini Imbulpitiya, Thamudika Pushpakumari

**Affiliations:** Department of Medicine, University of Peradeniya, Peradeniya, Sri Lanka; Teaching Hospital Peradeniya, Peradeniya, Sri Lanka

**Keywords:** Complementary and Alternative Medicine (CAM), Type 2 Diabetes mellitus, Bitter gourd, Crepe ginger, Ivy gourd, Fenugreek, Salacia reticulata

## Abstract

**Background:**

The incidence of chronic illnesses has increased worldwide. Diabetes is one such illness and 80% of the diabetic population lives in the developing world. There is a rapidly growing trend towards the use of Complementary and Alternative Medical practices in Diabetes. Sri Lanka is a developing Asian nation with a rich culture of Ayurvedic and native medical culture.

The objective of this study was to find the prevalence of use of CAMs in a diabetic population attending a large multiethnic diabetes facility in a University unit and to assess whether there is an increase in the incidence of hypoglycaemic episodes among users of CAMs.

**Methods:**

A cross sectional study was performed at Teaching Hospital Peradeniya between April and August 2012. Following verbal consent, 254 type 2 adult diabetic patients attending the diabetes facility were interviewed regarding the use of CAM and hypoglycaemia using an interviewer-administered questionnaire.

**Results:**

Of the 252 valid results, 192 patients (76%) admitted to the use of a CAM to reduce blood glucose. Bitter gourd, ivy gourd and crepe ginger were used by 128, 113 and 92 individuals. While 19% used a single agent, 34%, 21% and 2.4% used 2,3 and more than 3 agents. The incidence of hypoglycaemia in CAM users was 21% and 16.6% in non-users. The difference was statistically not significant. (p = 0.57) Ingestion of Costus speciosus (Crepe ginger) was associated with higher incidence of hypoglycaemia (P = 0.01).

Female gender was significantly associated with CAM use (p = 0.01), while the age, duration of diabetes, presence of co-morbidities and complications of diabetes failed to show a significant association.

**Conclusion:**

Sri Lanka has a very high use of herbal supplementation in type 2 diabetes patients. Although the overall incidence of hypoglycaemia is not increased among CAM users, crepe ginger is associated with significant hypoglycaemia and warrants further research.

**Electronic supplementary material:**

The online version of this article (doi:10.1186/1472-6882-14-374) contains supplementary material, which is available to authorized users.

## Background

### The burden of type2 diabetes

Diabetes mellitus is a major cause of morbidity and mortality worldwide with an increasing prevalence. The WHO estimates a prevalence of 347 million people with diabetes worldwide in 2013 [1]. The prevalence is expected to double between 2005–2030 and the greater proportion of this increase would be in the low to middle income countries of Asia, Africa and South America [2].

There is an emerging trend worldwide for patients to use complementary and alternative medications (CAM) in an attempt to improve the outcomes of their illnesses as well as to improve general well being. In addition, CAMs have gained academic, industrial and economic interest due to its high prevalence of use.

The National Centre for Complementary and Alternative Medicine of the United States defines CAM as “a group of medical and health care systems, practices and products that are not presently considered to be part of conventional medicine”. These agents seem to have become an attractive option because of the lesser-perceived adverse reactions in comparison to prescription medications. CAM incorporates herbal remedies and other forms of therapy like acupuncture, faith healing, massage therapy, hypnosis and music therapy [3].

Diabetes mellitus is an illness, where a wide array of CAMs has been used with varying success. Around 2–3.6 million people in the United States rely on complementary and alternative medicines for the treatment of their diabetes mellitus [4]. Recent estimates show that over 80% of people living in developing countries also still depend on CAM for treatment health conditions [4].

Medicinal plants, which are considered a prominent component of CAMs, still play a prominent role in human health care worldwide. During the last few years, even in the absence of good supporting evidence, the United States alone has recorded an increase of 380% in the use of herbal remedies [5].

Metformin, the first choice in the treatment of type 2 diabetes originated from the plant Galega offcinalis (French lilac or goats rue) and was once considered a CAM [6]. Over 400 plants and compounds have so far been evaluated for use in T2 diabetic patients and over 1200 have been claimed to be remedies for the same illness [7].

The worldwide trend for the use of CAMs in diabetes has increased with an overall prevalence ranging between 30%-57% in some studies [8]. Diabetic patients are 1.6 times more likely than non-diabetics to use a CAM for a host of reasons [9].

Australia and the United Kingdom records a prevalence of 46% among diabetics [10, 11].

India, a country that is steeped in tradition and boasting a rich history of healing practices records a very high use of 67% among its diabetic population. The majority of these patients (97%) used naturoapathy, which often included herbalism [3]. This brings about a hitherto unrecognized issue of using herbal remedies in conjunction with conventional medicines, raising the possibility of interactions and adverse drug reactions.

Cinnamon, garlic preparations and fenugreek and multi-vitamins are some of the popular over the counter CAMs used among diabetics [11].

The objective of this study was to evaluate the practice of Complementary and alternative Medicine among a group of Sri Lankan diabetics and to find out if those who use CAM in addition to conventional therapy are at a greater risk of hypoglycaemia.

## Methods

A cross-sectional survey was performed between April and August 2012 using an interviewer based questionnaire at the Diabetes clinic at Teaching Hospital Peradeniya, Sri Lanka. The questionnaire was designed following review of the traditional practices in the local community. Ethical review committee of the Faculty of Medicine, University of Peradeniya, Sri Lanka approved the project (2010/EC/49). A copy of the data collection from is appended. See Additional file 1.

A 2 stage sampling design was undertaken, with an initial pilot test being administered to 25 subjects to establish face validity and identify any questions that needed rephrasing. The questionnaire was then administered to 252 randomly selected type 2 diabetics attending the clinic for follow up visits from a total of 2250 registered patients. Questionnaire comprised of questions divided into the 3 domains of demographic data, diabetes related information and information on use of complementary and alternative medicine. (CAM) Demographic data included age, gender, occupation, the diabetes related information included duration of diabetes, presence of complications, current anti-diabetic medication, last reported Fasting Plasma Glucose (FPG) and HbA1C and the presence of hypoglycaemic episodes. CAM related information included the type of CAM and the reason for using it. The patients were specifically asked if these remedies were used as a CAM to supplement diabetes care, rather than an ordinary food item.

The inclusion criteria included a previous diagnosis of type 2 diabetes mellitus according to American Diabetes Association (ADA) criteria, age over 18 years and the ability to give verbal consent. Exclusion criterion was the inability to give consent due to physical, intellectual or psychiatric disease.

### Data analysis

The data analysis was conducted using the Statistical Package for the Social Sciences (SPSS, version 20) for Windows. A p value of 0.05 was used to determine any statistically significant difference between CAM and non-CAM users.

## Results

A total of 260 patients were approached and 254 patients consented to be included in the study. Data of 2 patients were removed as they contained incomplete data. The study population included 85males and 167 females with a mean age of 59.4 (SD: 9.55) years.

All patients were on oral Hypoglycaemic therapy. Six patients took insulin in combination with other oral hypoglycaemic agents. 223 patients were on Metformin alone or in combination with another oral agent. 27 patients were on oral therapy excluding Metformin.

192 patients (76.1%) used some form of herbal supplementation with the aim of reducing glucose values. 60 patients (23.9%) did not use any. Out of a total of 192 users, 53 (27%) were males and 139 (73%) females. Of the non-users, 32 (53%) were males and 28 (47%) females.

49 (19.4%) patients used a single agent, while 86 (34%), 52 (21%) and 6 (2.4%) patients used 2, 3 and more than 3 varieties of herbal supplementation to their meals respectively. 80% of the population used the supplementation at least once a week.

The duration of diabetes in the whole population was 8.4 years (SD 6.0), in CAM users 8.6 years (SD: 6.0) and in non-CAM users 7.7 years (SD: 5.7). The difference was not statistically significant. (P = 0.7) 161 patients reported the presence of at least one co-morbidity, which included the presence of Hypertension, Ischaemic heart disease or dyslipideamia. 143/192 CAM users and 18/60 non-CAM users reported the presence of a co-morbidity. This difference was not statistically significant. (P = 0.44) 27 out of 192 CAM users and 12 out 60 non-CAM users reported the presence of micro-vascular complications with a p value of 0.12.

Mean fasting plasma glucose of CAM users and non-users were 8.0 mmol/L (SD 3.2), and 7.27 mmol/L (SD 2.4) respectively. The difference was not statistically significant (p = 0.08).

The presence of hypoglycaemia was calculated by presence of any 3 of the following symptoms (hunger, sweating, palpitations, lightheadedness, confusion, loss of consciousness and seizures) relieved by carbohydrate intake. Overall, 51/252, (20.2%) experienced hypoglycaemia. Only 10/60, (16.6%) of non-CAM users experienced hypoglycaemia compared to 41/192 (21%) on herbal supplementation. There was no significant increase in the incidence of hypoglycaemia in CAM users. (p = 0.57).

The most commonly used CAM was bitter gourd (*Momordica charantia*) in 128 (50.8%) instances followed by ivy gourd (*Coccinia grandis*) leaves (113, 44.8%), crepe ginger (*Costus speciosus*) leaves (92, 36.5%), *Salacia reticulata* (17, 6.7%) and fenugreek (5, 2%) (Table 1).

**Table 1:** **Use of CAMs – frequency of use and the presence of hypoglycemic symptoms**

	CAM used	Frequency of use	Presence of hypoglycemia (%)	P. value
1	Momordica charantia (Bitter gourd)	128	21	0.73
2	Costus speciosus (Crepe ginger)	92	27.2	0.039
3	Coccinia grandis (Ivy gourd)	113	21.2	0.72
4	Fenugreek	5	40	0.26
5	Salacia reticulata	17	11.8	0.37

Bitter gourd was used as an accompanying curry with meals or as the blended fresh juice. The leaves of Costus speciosus and Coccinia grandis were consumed as a traditional leafy salad. The salad is made by shredding the leaves and then mixing it with condiments, grated coconut and salt. These leafy salads were an addition to the daily rice based meal. Fenugreek seeds were boiled and the water was consumed as herbal tea. Salacia reticulata was also boiled and the water was again consumed as herbal drink.

Binary logistic regression revealed a significant association between the use of Costus speciosus leaves and the incidence of hypoglycaemic episodes. (p = 0.039) None of the other CAMs showed a significant association with hypoglycaemia.

Female gender was significantly associated with CAM use (p = 0.01), while the age of the subject, duration of diabetes, and the presence of comorbidities and complications of diabetes failed to show a significant association (Table 2).

**Table 2:** **Association of age, gender, duration of diabetes, complications and co-morbidities with use of CAMs**

	Category	Using CAM	Not using CAMs	P. value
1	Age (Yrs.)	59.33	59.75	0.7
2	Duration of Diabetes (Yrs.)	8.6	7.7	0.7
3	Gender Male (No.)	53	32	0.4
4	Female (No.)	139	28	0.01
5	Presence of complications	27	12	0.12
6	Presence of co-morbidities	143	18	0.44

## Discussion

In this study we highlight a common practice among diabetic patients in Sri Lanka. The study was specifically focused to find out the non-commercially available types of CAMs used by diabetics as supplements to daily food intake. To the best of our knowledge this is the first study of its kind performed in Sri Lanka to assess the use of supplemental herbal remedies among diabetics on conventional treatment. The overall use of herbal medicine was very high (76%) among our study population. This is comparable to the study performed by kumar et al., which recorded an overall prevalence of 67% for CAMs among diabetic patients in India [3]. In this study there was a very high prevalence for the use of naturopathy (97%) comparable to our study. The United States and Mexico also have comparable figures of 73% and 62% respectively [8, 12]. However the prevalence for CAMs use in other countries was less, with both the United Kingdom and Australia recording 46% [10]. This difference in prevalence may be accounted for by the differences in definition of CAM and varying research designs. However most of these studies considered other forms of CAM such as ayurvedic medicine, acupuncture and physical methods when the prevalence was calculated.

Our study is unique in that it addressed the specific question of dietary herbal supplementation of type2 diabetics on conventional medicine. These supplements were used as non-proprietary traditional home-cooked dishes or tea. There are no other studies that described this type of dietary supplementation before.

It is important to note that all patients who used herbal supplementation were either on one or more oral hypoglycaemic agent, raising the possibility of drug interactions as well as potentiation of the hypoglycaemic effect of western medicine.

In our study, bitter gourd (momordica charantia) was the most frequently consumed herbal medication (128/193, 65.8%), followed by ivy gourd (coccinia grandis) 112/193, 58%, crepe ginger (costus speciosus) 91/193, 47%, and Fenugreek 5/193, 2.5% respectively. Singh et al. who studied the self-medication using herbal remedies in type 2 diabetics in India observed a high use of bitter gourd among the study population (51.1%) [13]. A direct comparison is possible between this study and ours because of common beliefs and practices between 2 closely linked populations as well as because of common geographical and ethno-botanical diversity.

Johnson et al. and Amireshani, in 2 similar surveys studied the use of herbal remedies among Hispanic diabetic immigrants in the United States. They observed a prevalence for use of herbal remedies in 91% and 77% of their respective study populations, in keeping with our findings [14, 15]. However, the type of herbal medicines consumed varied with the geographical location, which leads us to the conclusion that most of these practices are ethno-culturally centered.

In our study hypoglycaemic symptoms were experienced by 16.6% of patients on conventional medication, compared to 21% in patients on conventional medication and herbal supplements. 90% of patients experiencing hypoglycaemia used 2 or more herbal supplements. However, these findings did not reach statistical significance.

The increased incidence of hypoglycaemic symptoms may be a direct action of the herbal remedies or a potentiation of the hypoglycaemic effect of the conventional medicine. Hypoglycaemic effect of both bitter gourd and ivy gourd has been studied in humans and bitter gourd may possess an acute hypoglycaemic effect following ingestion [16, 17].

Cinnamon and fenugreek are 2 other herbal remedies that have gained popular usage among diabetics with proven efficacy in human studies. However in our study, fenugreek use was very low (5/192) and cinnamon was not used at all.

We demonstrated a significant association of hypoglycaemia (p = 0.039) with the use of *Costus speciosus* leaves. Although there are reported instances of use of its rhizome for hypoglycaemic effects, [18, 19] this is the first instance that the use of its leaves is documented. In view of the significant association with hypoglycaemia there is an urgent need to study this in finer detail.

Use of CAMs was significantly associated with the female gender, but not with the age of the patient, duration of diabetes, presence of co-morbidities or complications of diabetes.

There is trend towards the use of herbal supplementation and the presence of complications, but this did not reach statistical significance. This effect may be seen if a larger sample had been used.

The measurement of hypoglycaemia, based on a symptom score rather an objective biochemical value, non-availability of HbA1c measurements, and a small sample size are identified as limitations in this study, which should be addressed subsequent studies.

## Conclusion

Sri Lanka has a very high prevalence for the use of herbal dietary supplementation. General use of CAM does not increase the incidence of hypoglycaemia, but the leaves of *Costus speciosus* is significantly associated with a higher incidence of hypoglycaemic episodes and warrants further research.

## Electronic supplementary material

Additional file 1: **Questionnaire on use of CAM and symptoms of hypoglycaemia.** (DOC 260 KB)

## Abbreviations

CAM: Complementary and alternative medicine.
