# Supplementary material for: Use of Complementary and Alternative Medicines (CAMs) among type 2 diabetes patients in Sri Lanka: a cross sectional survey
Source: BMC Complement Altern Med. 2014 Oct 4;14:374. doi: 10.1186/1472-6882-14-374 (PMC4201716; doi:10.1186/1472-6882-14-374)
Supplement: Supplementary file 1 — Additional file 1: Questionnaire on use of CAM and symptoms of hypoglycaemia. (DOC 260 KB) [file 12906_2014_1964_MOESM1_ESM.doc]

**Questionnaire on use of CAM and symptoms of hypoglycaemia**

**Demographics**

Optional

1. Name

DD / MM / YYYY

1. Age:
2. Gender Male Female
3. Marital status : Married Unmarried
4. Residence: ___________________________________________________

___________________________________________________

1. Occupation 1.Student 2.Unemployed

3.Retired 4.State Occupation

1. Monthly income ( in Rs)

………………………………………….

1. Highest educational qualification: 1. Up to O/L 2. O/L 3. A/L 4. Graduate/Post graduate
2. Religion : B /C /M /H / Other
3. Height cm Weight kg

**Diabetes:**

1. Duration of diabetes (………….yrs…………….months)
2. Current diabetes Medications:
   1. Insulin
   2. Metformin
   3. Glibenclamide
   4. Tolbutamide
   5. Glitazones
   6. Gliclazide
   7. Glimipiride
   8. Other
3. Complications at present
   1. Retinopathy : Yes No
   2. Nephropathy: Yes No
   3. Neuropathy: Yes No
   4. IHD : Yes No
   5. CVA: Yes No
   6. PVD: Yes No
   7. Sexual dysfunction : Yes No

DD / MM / YYYY

1. The Last available HbA1c………………………………………….. Date ………………………..
2. The last available FBS…………………………………………………Date …………………………

DD / MM / YYYY

1. The last available PPBS……………………………………………….Date …………………………..

**CAM:**

1. Have you ever used any form of CAM?

Yes, at present Yes, in the past ( Go to 4) No ( Go to 14)

1. Type of CAM at present
   1. Herbal Therapy: / traditional medicines (state)

| Name | Preparation | Quantity | Frequency |
| --- | --- | --- | --- |
|  |  |  |  |

b. Meeting a non conventional physician

Ayurvedic Homeopathic Yunani

Other: Frequency …………………………………………

c. Religious healing therapy …………………………………………………………..

d. Yoga ………………………………………………………….

e. Exercises …………………………………………………………..

f. Other

1. For what perceived purpose do you take it?

1.improving blood sugar control 2.improve general well being 3.sexual dysfunction 4.other specific reason

If you ever discontinued a CAM

1. What is the CAM? ……………………..
2. What is the reason for discontinuing? …………………………..
3. Have you had any side effects from CAMs?

Yes No

If yes state what ……………………………….………………………………………

7. Do you think CAM improved your general well being?

Yes No

8. Do you think CAM improved your blood sugar control?

Yes No

9.From where did you learn about the CAM?

Doctor Another health care worker Media

Another patient Family member Friend

Alternative practitioner

10.Who recommended the CAM?

Doctor Another Healthcare worker Media Another patient Family member Friend

Alternative practitioner

1. Have you had any of the following symptoms during last 3 months at any one time?

| Symptom | 1^st^ | | | 2^nd^ | | | 3^rd^ | | | 4^th^ | | |
| --- | --- | --- | --- | --- | --- | --- | --- | --- | --- | --- | --- | --- |
|  | Yes | No | * | Yes | No | * | Yes | No | * | Yes | No | * |
| Palpitations |  |  |  |  |  |  |  |  |  |  |  |  |
| Tremor |  |  |  |  |  |  |  |  |  |  |  |  |
| Sweating |  |  |  |  |  |  |  |  |  |  |  |  |
| Faintishness/Dizziness |  |  |  |  |  |  |  |  |  |  |  |  |
| Visual disturbance |  |  |  |  |  |  |  |  |  |  |  |  |
| Confusion |  |  |  |  |  |  |  |  |  |  |  |  |
| Seizures |  |  |  |  |  |  |  |  |  |  |  |  |
| LOC |  |  |  |  |  |  |  |  |  |  |  |  |
| Other |  |  |  |  |  |  |  |  |  |  |  |  |

* Relieved by a sugary drink or snack

If you had LOC, state the number of times (1, 2, 3 etc. ) ……………………………………..

Where did you go for treatment? ……………………………………………………………………...

12. Does your regular or clinic doctor know about your CAM use?

Yes, He asked me Yes, I told him No

13. From where do you get your CAM? Local source Paid source

14. How much money do you spend for a week on CAM? (in Rs/Week) .........................

15. Have you visited an alternative Medical Practitioner for your Diabetes during the last 3 months? Yes No

16. If you have never used a CAM before, would you consider using one in future?

Yes No
